# Regulatory Effect of Polyacrylate Emulsion on the NaCl Attack Behavior of Cement-Based Grouting Materials

**DOI:** 10.3390/polym18172039

**Published:** 2026-08-22

**Authors:** Yuxuan Wang, Shengjie Han, Fan Wang, Lei Hu, Jiao Liao, Shijie Zhu, Yangyang Li, Jiehao Wu

**Affiliations:** 1 School of Civil Engineering and Architecture, Anhui University of Science and Technology, Huainan 232001, China; wangyx95@foxmail.com (Y.W.); hansj420@126.com (S.H.); wf2947115691@163.com (F.W.); hlydky@163.com (L.H.); 13365876231@163.com (J.L.); sjzhum@163.com (S.Z.); 15515510172@163.com (Y.L.); 2 Zhiruiyuan Traffic Consultation Limited Company, Chongqing 400030, China

**Keywords:** polyacrylate emulsion, cement-based grouting material, NaCl attack, pore structure, Friedel’s salt, mechanical strength retention

## Abstract

Cement-based grouting materials with a high water-to-cement ratio are susceptible to connected pore development, chloride ingress, and mechanical degradation in chloride-bearing groundwater and marine environments. To improve resistance to NaCl attack, this study compared an unmodified cement-based grouting material (NC) with a polyacrylate-emulsion-modified material (PA). Mechanical properties, surface wettability, pore structure, phase assemblage, thermal behavior, functional groups, and microstructure were investigated under different NaCl concentrations (0%, 5%, 10%, and 15%) and immersion durations (28 and 90 d). This study systematically evaluates the coupled evolution of mechanical strength retention, surface wettability, pore structure, chloride-bearing phases, and microstructure in a bulk PA-emulsion-modified high-water-to-cement-ratio grouting material under graded NaCl exposure. The results showed pronounced concentration- and time-dependent effects. Low NaCl concentrations were associated with continued hydration and reaction-product filling, whereas higher concentrations and prolonged exposure led to pore coarsening and strength loss. PA modification improved the mechanical stability of the material in NaCl environments. After 90 d of immersion in 15% NaCl, the compressive and flexural strengths of the PA group were 23.60% and 22.53% higher than those of the NC group, respectively, while the corresponding strength-retention ratios were higher by 9.06 and 10.40 percentage points. Contact-angle and MIP results showed that PA reduced surface wettability and mercury-accessible porosity. After 15% NaCl exposure, the contact angle of the PA group remained 72.5°, compared with 40.1° for the NC group, while the porosity decreased from 38.11% in the NC group to 30.98% in the PA group. XRD, TG-DTG, and FTIR analyses indicated the formation and evolution of Friedel’s salt or other chloride-bearing AFm phases after NaCl exposure. Combined with SEM observations, the results indicate that PA mitigates NaCl-induced deterioration through reduced surface wettability, refined pore structure, regulated chloride-bearing product distribution, and improved matrix integrity. Overall, the findings establish a coupled surface–pore–phase–microstructure framework for understanding the enhanced NaCl resistance of PA-modified cement-based grouting materials.

## 1. Introduction

Cement-based grouting materials are widely used for underground engineering reinforcement, crack sealing, foundation treatment, and leakage control because their fluidity can be adjusted, they adapt well to construction conditions, and they develop relatively high load-bearing capacity after hardening. The injectability of grout is jointly controlled by particle size, rheological behavior, filtration effects, and resistance within porous media. A relatively high water-to-cement ratio is therefore commonly used to allow grout to penetrate fine cracks [1,2]. However, high water content readily produces capillary pores, connected pores, and interfacial defects in the hardened body, providing pathways for groundwater and salt solutions to enter the material. When grouting materials serve in coastal areas, saline soils, or chloride-rich groundwater, NaCl solution can enter the matrix through the pore network and continuously alter the pore solution chemistry, hydration-product stability, and internal transport conditions. Therefore, deterioration of cement-based grouting materials in chloride environments depends not only on the external NaCl concentration and exposure duration, but also on the initial pore structure, surface wettability, and crack-propagation resistance.

After NaCl enters the cement matrix, Cl^−^ can remain in the pore solution, be adsorbed on the surface of hydration products such as C-S-H, or chemically bind with Al-bearing AFm phases [3,4]. During this process, Cl^−^ can participate in the formation of Kuzel’s salt, Friedel’s salt, and other chloride-bearing AFm phases, while also altering the composition and structure of the original AFm phases [5,6]. The degree of chloride binding is affected by the hydration-product assemblage, external salt concentration, pore-solution environment, and associated cation type. AFm phases mainly contribute to chemical binding, whereas C-S-H primarily contributes to physical adsorption [7,8]. Previous studies have further shown that Friedel’s salt represents only part of the bound chloride, and that its amount and spatial distribution are jointly constrained by the availability of Al-bearing phases, ion-migration conditions and local calcium leaching [9]. NaCl exposure also changes pore-size distribution and mercury-accessible pore volume, thereby coupling chloride transport, reaction-product deposition and pore-structure evolution [10]. At lower concentrations or shorter exposure times, continued hydration and deposition of chloride-bearing products may fill some pores. As NaCl concentration and exposure duration increase, salt enrichment, product rearrangement, and local crystallization pressure may promote pore coarsening and mechanical degradation [11]. Under high NaCl concentrations and specific temperature conditions, unfavorable hydration-phase transformations and secondary ettringite formation may also occur [12,13]. Thus, the effect of NaCl on non-reinforced cement-based materials should not be reduced to chloride penetration alone, but should be understood as a dynamic process involving solution transport, chloride binding, solid-phase transformation, pore filling, and structural damage.

Polymer modification can simultaneously affect hydration, pore structure, interfacial bonding, and the transport of aggressive media in cement-based materials. During cement hydration and water consumption, emulsion particles gradually aggregate and may distribute within interparticle gaps, capillary pores, and hydration-product interfaces, forming local or continuous polymer phases under suitable conditions [14]. The organic phase can reduce the connectivity of some open pores, bridge hydration products, and improve interfacial deformation compatibility. However, its actual effect depends on polymer type, dosage, film-forming conditions, and curing environment. Long-term wet curing may lead to water absorption and swelling of the polymer film, thereby readjusting the microstructure and mechanical properties [15]. In PA-modified systems, polymer particles can adsorb on cement particles and hydration products, changing the distribution of hydration products and the continuity of the matrix [16]. The highly alkaline pore solution may also promote partial ester hydrolysis, and the resulting carboxyl groups can coordinate or crosslink with Ca^2+^, strengthening the connection between the organic phase and inorganic hydration phases [17,18]. Appropriate amounts of acrylic emulsion or other polymers generally reduce water absorption and the transport of aggressive media while improving flexural performance and crack-propagation resistance [19,20]. In chloride environments, polymers such as SBR, PAE, and VAE can slow Cl^−^ ingress by refining the pore structure, reducing pore connectivity, and increasing ion-transport resistance, although the effect depends strongly on polymer type, curing regime, and exposure condition [21,22]. Polymers may also indirectly affect chloride binding and chloride-bearing product distribution by changing the stability of Al-bearing hydration products and the migration rate of Cl^−^. Recently, modified PA emulsions have been used to improve the hydrophobicity and impermeability of cement-based coatings [23], and acrylamide in situ polymerization systems have also shown good stability under chloride immersion and chloride-binding capacity [24]. Nevertheless, coating systems, in situ polymerization systems, and bulk emulsion-modified systems differ substantially in polymer content, film-forming mode, and exposure boundary conditions; therefore, existing conclusions cannot directly explain the response of PA-emulsion-modified ordinary Portland cement-based grouting materials under long-term NaCl immersion.

A previous study [25] showed that PA can reduce the porosity of cement-based grouting materials, improve the connection between hydration products, and mitigate pore coarsening and crack propagation under sulfate attack. However, sulfate attack is mainly governed by the accumulation of expansive products such as gypsum and ettringite, whereas NaCl environments involve free-chloride migration, C-S-H surface adsorption, AFm phase transformation, and Friedel’s salt formation. The reaction pathways and damage characteristics of the two salt-exposure conditions are substantially different. Although many studies have reported the mechanical enhancement, film formation, and impermeability improvement of PA-modified cement-based materials, systematic correlations among surface wettability, pore structure, chloride-bearing phase evolution, and crack development under the combined effects of NaCl concentration and immersion duration remain insufficient for high-water-to-cement-ratio grouting materials. In particular, it remains necessary to clarify whether PA changes the formation state of chloride-bearing products such as Friedel’s salt while reducing solution wetting and pore connectivity, and how such changes affect long-term mechanical stability. Accordingly, this study used unmodified and PA-modified cement-based grouting materials, with 0%, 5%, 10%, and 15% NaCl solutions and immersion durations of 28 d and 90 d. Compressive strength, flexural strength, and strength retention were measured, and contact angle, MIP, XRD, TG-DTG, FTIR, and SEM were used to analyze changes in surface wettability, pore structure, phase assemblage, and microstructure. The aim was to clarify the combined effects of PA on NaCl-solution ingress, Cl^−^ migration and binding, attack-product distribution, and microcrack propagation, and to reveal the competition between hydration/product filling at low concentrations and structural damage under high-concentration and long-term exposure. The results provide a basis for durability design of PA-modified cement-based grouting materials in saline groundwater and coastal engineering.

## 2. Materials and Methods

### 2.1. Raw Materials

P·O 32.5 ordinary Portland cement supplied by Anhui Conch Cement Co., Ltd. (Wuhu, China) was used as the cementitious material. The cement type and grade were identified according to the manufacturer’s product specification, and its major oxide composition is presented in Table 1. The measured contents of CaO, SiO_2_, Al_2_O_3_, MgO, Fe_2_O_3_, and SO_3_ were 63.00%, 21.23%, 6.58%, 1.60%, 3.65%, and 2.93% by mass, respectively.

The polyacrylate emulsion (PA) was a commercial waterborne polymer emulsion supplied by Shandong Yousuo Chemical Technology Co., Ltd. (Linyi, China) The emulsion was used as received. Its basic properties are summarized in Table 2: it was a milky white liquid with a pH of 7.0–9.0, a viscosity of 100–500 mPa·s, and a solids content of approximately 76%.

Analytical-grade sodium chloride was used to prepare the NaCl exposure solutions. Deionized water was used for mixing, preparation of the NaCl solutions, and water-immersion controls. No aggregate, sand, mineral filler, accelerator, or other chemical admixture was incorporated into either the NC or PA formulation.

### 2.2. Mix Design

Previous work on similar polymer-modified grouting materials showed that a PA-emulsion dosage of 12% by mass of cement provided a favorable balance between grout fluidity and hardened mechanical properties [25]. Therefore, unmodified ordinary cement-based grouting material was used as the control group (NC), and material modified with 12% PA was used as the modified group (PA). The present study focused on the NaCl-resistance behavior of this fixed 12% PA-emulsion formulation, and the conclusions regarding PA modification refer to the dosage investigated here. P·O 32.5 ordinary Portland cement was used in both mixtures, and no additional chemical admixtures were incorporated. The mass ratio of cement to total water was maintained at 1:0.8. The water contained in the PA emulsion was included in the total water content and deducted from the added water accordingly. The mix proportions are listed in Table 3. The PA dosage was calculated using the wet emulsion mass; based on the measured solids content of 76%, the corresponding dry polymer-to-cement ratio was 9.12%. To eliminate the influence of variations in emulsion water content on the effective water-to-cement ratio, the amount of added water in each batch was corrected according to the measured solids content of the emulsion.

### 2.3. Specimen Preparation

**Figure 1:**
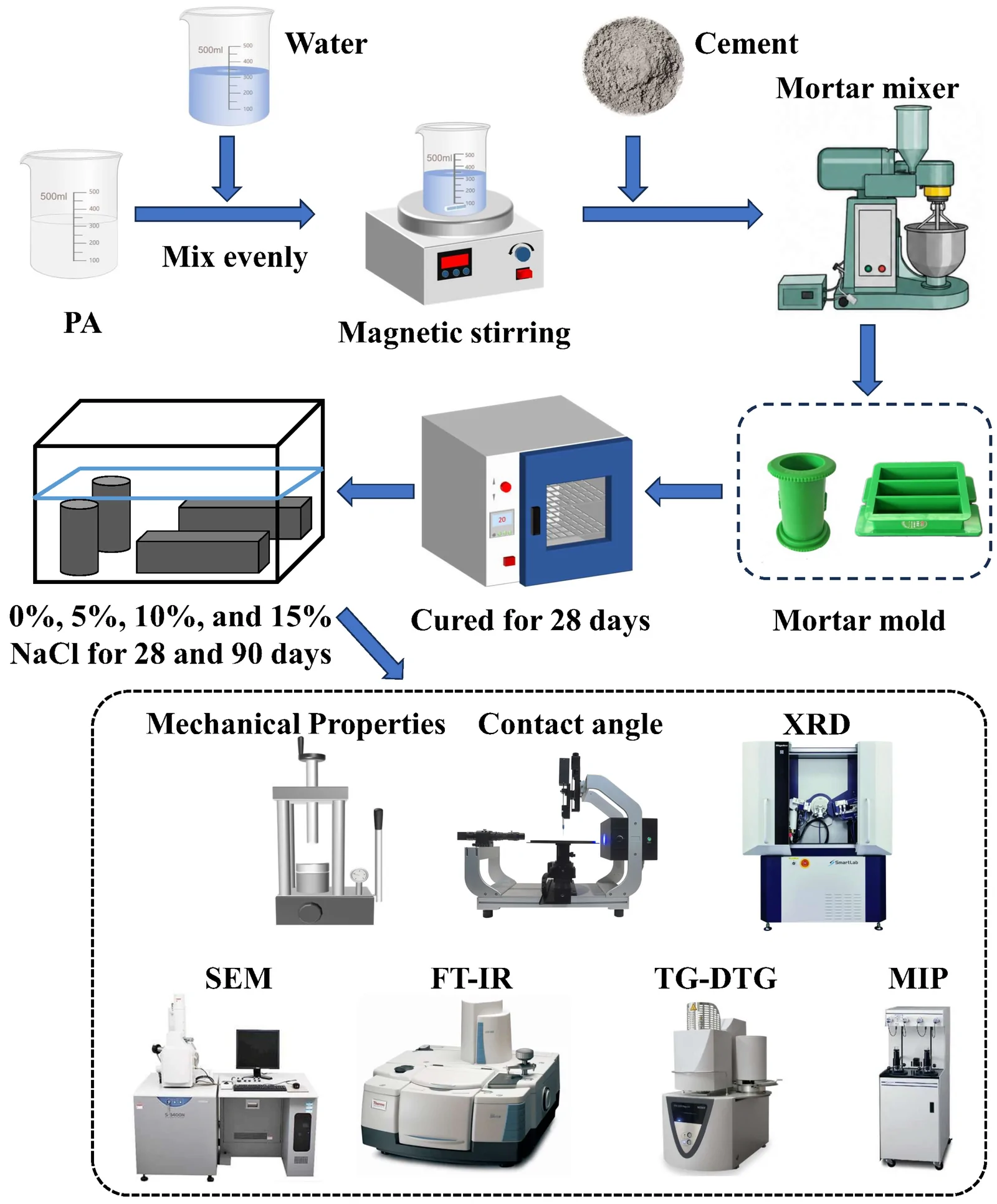
Schematic diagram of specimen preparation, NaCl immersion, and performance characterization.

First, the PA emulsion was mixed with the corrected mixing water to disperse the emulsion uniformly in the aqueous phase. The NC mixture contained no PA emulsion and was prepared with the corresponding amount of mixing water. Cement was then mixed with the liquid phase under continuous stirring. After low-speed dispersion and high-speed mixing until homogeneous, the slurry was immediately cast into molds. Slight vibration or manual rodding was used to remove large bubbles, and the specimens were covered with plastic film to reduce early moisture loss. The specimen preparation, immersion, and testing procedure is shown in Figure 1.

The specimens were demolded after 24 h and cured for 28 d under standard curing conditions of 20 ± 2 °C and relative humidity not lower than 95%. Cylindrical specimens with a diameter of 50 mm and a height of 100 mm were used for compressive-strength tests, and 40 mm × 40 mm × 160 mm prisms were used for flexural-strength tests. Samples for contact-angle, MIP, XRD, TG-DTG, FTIR, and SEM analyses were taken from specimens in the same batches. Three parallel specimens (*n* = 3) were tested for each mechanical-property condition.

### 2.4. NaCl Immersion Regime

After standard curing, NC and PA specimens were immersed in NaCl solutions with mass fractions of 0%, 5%, 10%, and 15%. The concentrations of 5%, 10%, and 15% were selected to establish a progressively increasing NaCl-exposure gradient, allowing the concentration-dependent response of the materials to be evaluated from relatively mild to severe chloride conditions. This range was intended to distinguish the potential hydration and reaction-product-filling effects at relatively low NaCl concentrations from the pore-structure deterioration and mechanical degradation that may become dominant at higher concentrations. The 0% NaCl group was immersed in deionized water and used as the same-age immersion reference. Water immersion maintained comparable moisture and exposure conditions while accounting for continued hydration, thereby allowing the specific influence of NaCl to be distinguished from the effects of specimen age and prolonged wet curing.

The immersion durations were selected as 28 d and 90 d to evaluate the time-dependent response of the materials at two laboratory exposure stages. The 28 d period was used to characterize the relatively early-to-intermediate response to NaCl after standard curing, whereas the 90 d period provided an extended exposure interval over which cumulative chloride ingress, hydration-product transformation, pore-structure evolution, and mechanical degradation could become more evident. These durations were selected for comparative laboratory evaluation and should not be regarded as direct equivalents of specific field service periods, because actual deterioration rates are also influenced by environmental factors such as chloride concentration, temperature, wetting–drying conditions, and groundwater transport. The solutions were prepared using analytical-grade NaCl and deionized water. Specimens were fully submerged in containers with appropriate spacing to ensure sufficient contact between all surfaces and the attack medium.

During immersion, the ambient temperature was maintained at 20 ± 2 °C. To reduce the effects of water evaporation and ion consumption on solution concentration, the solutions were replaced every 7 d with freshly prepared solutions of the same concentration, and evaporative losses were replenished regularly. The immersion durations were 28 d and 90 d. At the specified age, the specimens were removed, surface salts were rapidly rinsed off with deionized water, free surface water was wiped away with absorbent paper, and mechanical testing and microstructural characterization (90 d samples) were carried out under a consistent state.

For sample identification, groups were named according to material type and immersion medium, such as NC-Water, PA-Water, NC-15%NaCl, and PA-15%NaCl. PA samples exposed to different NaCl concentrations were denoted PA-5%NaCl, PA-10%NaCl, and PA-15%NaCl.

### 2.5. Mechanical Property Tests

Compressive strength was measured with reference to GB/T 50081-2019 [26], using a compression testing machine at a loading rate of 0.5 MPa/s until failure. Flexural strength was measured with reference to GB/T 17671-2021 [27], by three-point bending at a loading rate of 0.05 MPa/s. For each material type, NaCl concentration, and immersion duration, three parallel specimens (*n* = 3) were tested separately for compressive and flexural strength. The results are reported as the mean ± standard deviation (SD), and error bars represent one SD.

Statistical analyses were performed using the raw mechanical-property data. For each immersion duration (28 and 90 d), compressive and flexural strengths were analyzed separately by two-way analysis of variance (ANOVA), with material type (NC or PA) and NaCl concentration (0%, 5%, 10%, or 15%) as fixed factors. Šídák’s multiple-comparison test was used to compare NC and PA at each NaCl concentration. Differences were considered statistically significant at *p* < 0.05.

To evaluate the mechanical stability of the materials under different NaCl concentrations while accounting for continued hydration during immersion, compressive- and flexural-strength retention ratios were calculated using the corresponding same-material, same-age water-immersed specimens as references, as follows:

(1) $$R_{c}=\frac{f_{c,n,t}}{f_{c,0,t}}\times 100\%$$

(2) $$R_{f}=\frac{f_{f,n,t}}{f_{f,0,t}}\times 100\%$$

where $R_{c}$ and $R_{f}$ are the compressive- and flexural-strength retention ratios, respectively; $f_{c,n,t}$ and $f_{f,n,t}$ are the compressive and flexural strengths after immersion for t days in a NaCl solution of mass fraction *n*; and $f_{c,0,t}$ and $f_{f,0,t}$ are the corresponding strengths of the same material after water immersion for the same duration. Using the same-material, same-age water-immersed group as the reference reduces the influence of continued hydration and age on strength evaluation. Error bars in the figures represent one standard deviation.

### 2.6. Contact-Angle Test

Static water contact angles were measured using an FCA500F contact-angle meter (Shanghai AFES Precision Instrument Co., Ltd., Shanghai, China) for NC and PA specimens exposed to water and 15% NaCl solution. Specimens were cut from the same position, and the test surfaces were ground at low speed to obtain relatively flat and comparable surfaces. The samples were then dried at 40 °C to constant mass and cooled to room temperature in a desiccator. Surface wettability was measured by the sessile-drop method. Deionized water was used as the test liquid, with a single droplet volume of 5 μL. After the droplet contacted the specimen surface, its profile was captured within the specified time, and the contact angle was calculated by identifying the baseline and contact points.

### 2.7. Mercury Intrusion Porosimetry

The pore-size distribution, total mercury intrusion, and mercury-accessible porosity of different specimens were measured using a Micromeritics AutoPore V 9600 automatic mercury intrusion porosimeter (Micromeritics Instrument Corporation, Norcross, GA, USA). The pressure range was 0.10–61,000 psia, and the mercury contact angle was set to 130°. Total mercury intrusion, pore-size distribution, and porosity were used to evaluate pore-structure coarsening or filling after NaCl attack and to compare the regulation of pore connectivity and pore-size distribution by PA modification. It should be noted that MIP characterizes mercury-accessible pore entrances rather than the complete three-dimensional pore geometry; therefore, the calculated pore sizes are more appropriately interpreted as equivalent pore-entry or pore-throat diameters. The results may also be influenced by ink-bottle effects, pore connectivity, assumptions regarding pore geometry and mercury contact angle, and possible microstructural alteration under very high intrusion pressures. Accordingly, the MIP data in this study were primarily used for relative comparison of mercury-accessible pore structure among specimens tested under identical conditions rather than as an absolute representation of the true pore-size distribution.

### 2.8. X-Ray Diffraction Analysis

X-ray diffraction (XRD) was used to analyze changes in the main crystalline phases before and after exposure. A SmartLab diffractometer (Rigaku Corporation, Akishima, Tokyo, Japan) with Cu Kα radiation was used, with an operating voltage of 40 kV and current of 30 mA. The scanning range was 5–90° (2θ), the step size was 0.02°, and the scanning rate was 5°/min. Changes in characteristic peaks of portlandite, calcite, quartz, and Friedel’s salt were compared among specimens to evaluate the effects of NaCl attack and PA modification on hydration products and chloride-binding products.

### 2.9. Thermogravimetric Analysis

Thermogravimetry and derivative thermogravimetry (TG-DTG) were used to investigate the thermal decomposition behavior of bound water, calcium hydroxide, and calcium carbonate in the specimens. A NETZSCH STA 2500 thermal analyzer (NETZSCH-Gerätebau GmbH, Selb, Germany) was used. Samples were placed in Al_2_O_3_ crucibles and heated from 30 °C to 1000 °C under a nitrogen atmosphere at a heating rate of 20 °C/min. Mass losses in different temperature ranges and changes in DTG peaks were used to analyze C-S-H gel dehydration, Ca(OH)_2_ dehydroxylation, and CaCO_3_ decomposition, and to assist in interpreting the effects of NaCl attack and PA modification on hydration-product stability.

### 2.10. Fourier Transform Infrared Spectroscopy

Fourier transform infrared spectroscopy (FTIR) was used to characterize changes in the main chemical bonds and functional groups. The test was performed in absorbance mode using a SHIMADZU IRTracer-100 spectrometer (Shimadzu Corporation, Kyoto, Japan). The wavenumber range was 4000–400 cm^−1^, the resolution was 4 cm^−1^, the number of scans was 16, and a Happ-Genzel apodization function was used. Changes in O-H, H-O-H, CO_3_^2−^, Si-O, and PA-related carbonyl/organic functional-group absorption bands were emphasized to assist in identifying the evolution of hydration products, carbonate phases, and PA-modified components after exposure.

### 2.11. Scanning Electron Microscopy

Scanning electron microscopy (SEM) was used to observe the microstructure of specimens after exposure. An S-3400 scanning electron microscope (Hitachi High-Technologies Corporation, Tokyo, Japan) was used in secondary-electron mode with an accelerating voltage of 20 kV. Magnifications of ×1000, ×2000, and ×5000 were selected to observe matrix compactness, pore connectivity, crack development, and attack-product morphology. By comparing the microstructures of NC and PA groups, the effects of PA modification on product filling, pore bridging, and matrix integrity were analyzed.

## 3. Results and Discussion

### 3.1. Mechanical Property Evolution

**Figure 2:**
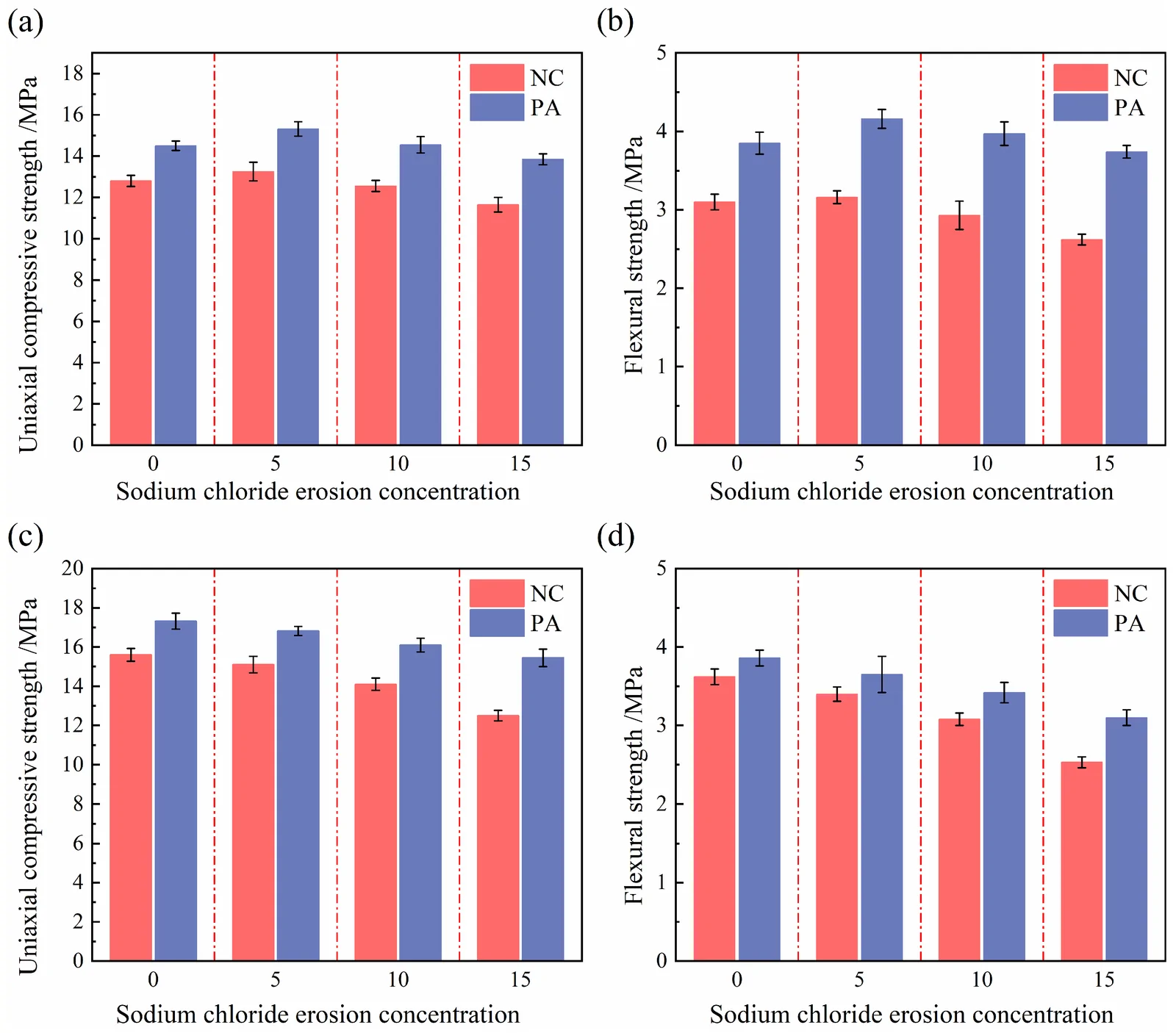
Mechanical strengths of NC and PA specimens under different NaCl concentrations and immersion durations: (**a**) 28 d compressive strength; (**b**) 28 d flexural strength; (**c**) 90 d compressive strength; (**d**) 90 d flexural strength.

Figure 2 shows the compressive and flexural strengths of the NC and PA groups under different NaCl concentrations and immersion durations. Overall, the mechanical properties of both groups were jointly affected by NaCl concentration and immersion time, and the PA-modified group exhibited higher mean strengths than the NC group under all test conditions.

Two-way ANOVA showed significant main effects of both material type and NaCl concentration on compressive and flexural strengths at both 28 and 90 d (all *p* < 0.0001). The material type × NaCl concentration interaction was significant for the 90 d compressive strength (F(3,16) = 4.107, *p* = 0.024), but was not significant for the 28 d compressive strength (*p* = 0.625), 28 d flexural strength (*p* = 0.091), or 90 d flexural strength (*p* = 0.102).

As shown in Figure 2a,c, the effect of NaCl concentration on compressive strength became more pronounced with increasing immersion duration. At 28 d, the compressive strengths of the NC group in 0%, 5%, 10%, and 15% NaCl solutions were 12.80, 13.25, 12.55, and 11.65 MPa, respectively, whereas those of the PA group were 14.50, 15.32, 14.55, and 13.85 MPa, corresponding to increases of 13.28%, 15.62%, 15.94%, and 18.88% relative to the NC group. Under 5% NaCl, the compressive strengths of both groups were slightly higher than those of the corresponding same-age water-immersed specimens. Because ongoing hydration was accounted for using the water-immersed reference, this increase represents a net effect associated with low-concentration NaCl exposure. The increase may be related to chloride-induced changes in hydration kinetics and/or the filling of pores by chloride-bearing or other reaction products. However, when the NaCl concentration increased to 10% and 15%, the compressive strength decreased relative to the water reference, indicating that deterioration gradually became dominant.

After the immersion duration was extended to 90 d, the compressive-strength loss caused by increasing NaCl concentration became more pronounced. The compressive strength of the NC group decreased from 15.60 MPa under 0% NaCl to 12.50 MPa under 15% NaCl, whereas that of the PA group decreased from 17.32 MPa to 15.45 MPa. Although both materials lost strength, the PA-modified group still maintained a higher strength under high-concentration NaCl. Under 15% NaCl for 90 d, the compressive strength of the PA group was 23.60% higher than that of the NC group, exceeding the improvement at 28 d under the same concentration. This indicates that PA modification more effectively inhibited compressive-strength degradation during long-term chloride attack.

Šídák-adjusted multiple comparisons showed that the compressive strength of the PA group was significantly higher than that of the NC group at all four NaCl concentrations at both 28 and 90 d (all adjusted *p* < 0.0001). The significant material type × NaCl concentration interaction observed at 90 d further indicates that the magnitude of the PA-associated improvement in compressive strength depended on NaCl concentration. Notably, under 15% NaCl for 90 d, the compressive strength of the PA group was 23.60% higher than that of the NC group.

Figure 2b,d shows the effect of NaCl attack on flexural strength. Compared with compressive strength, flexural strength was more sensitive to changes in NaCl concentration. At 28 d, the flexural strength of the NC group changed from 3.10 MPa under 0% NaCl to 2.62 MPa under 15% NaCl, whereas that of the PA group changed from 3.85 MPa to 3.74 MPa. The flexural strength of the PA group was higher than that of the NC group at all NaCl concentrations, and the advantage increased with NaCl concentration. Under 15% NaCl, the flexural strength of the PA group was 42.75% higher than that of the NC group, demonstrating that PA substantially improved flexural mechanical performance under high-concentration NaCl exposure. At 90 d, flexural strength decreased continuously with increasing NaCl concentration. The PA group exhibited higher mean flexural strengths than the NC group, although the differences were not statistically significant under water immersion (adjusted *p* = 0.109) or 5% NaCl (adjusted *p* = 0.096). Significant differences emerged under 10% NaCl (adjusted *p* = 0.014) and 15% NaCl (adjusted *p* < 0.001). Under 15% NaCl, the flexural strength of the NC group decreased to 2.53 MPa, whereas that of the PA group remained 3.10 MPa, 22.53% higher than that of the NC group. This indicates that the flexural-performance advantage associated with PA modification became particularly evident at higher NaCl concentrations after prolonged exposure. The higher flexural strength of the PA group, together with the denser and more continuous matrix subsequently observed by SEM, is consistent with better preservation of structural integrity under NaCl exposure.

**Figure 3:**
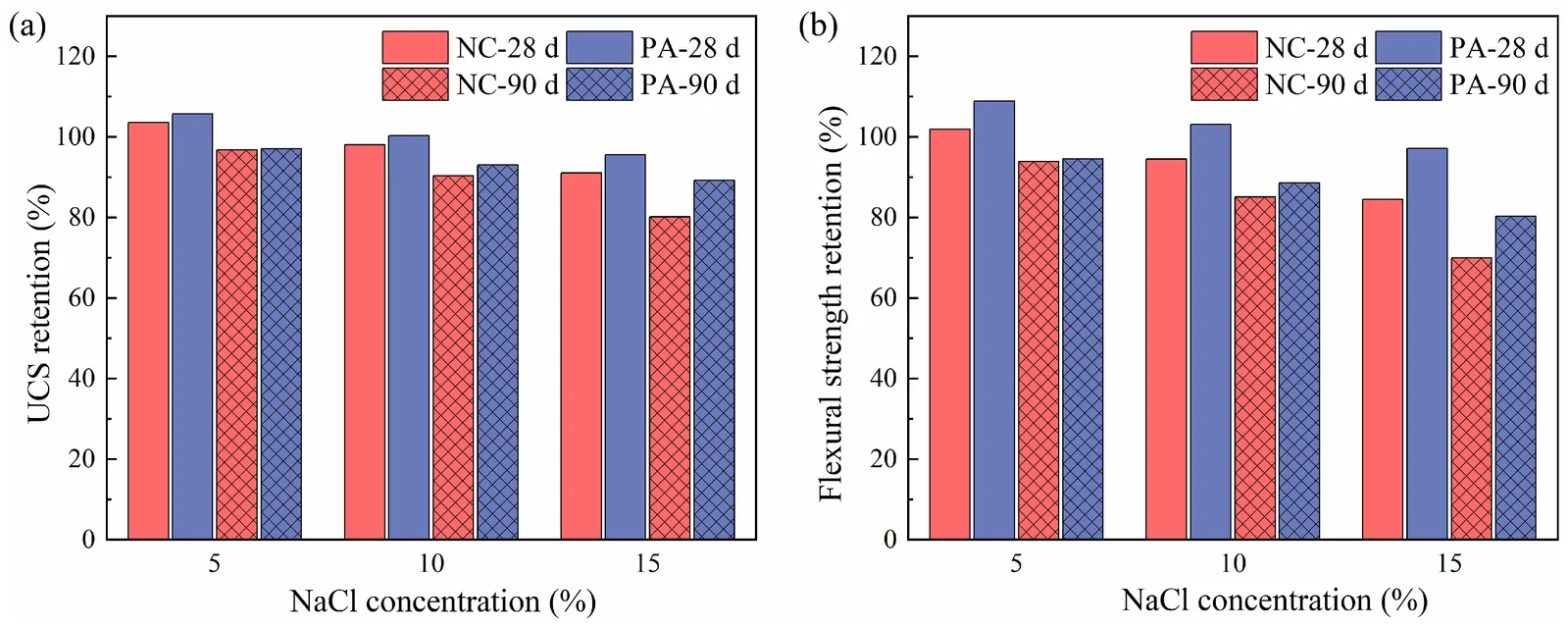
Strength retention of NC and PA specimens under different NaCl concentrations and immersion durations: (**a**) compressive strength retention; (**b**) flexural strength retention.

Figure 3a shows the variation in compressive strength retention under different NaCl concentrations and immersion durations. At 28 d, the compressive strength retentions of the NC group under 5%, 10%, and 15% NaCl were 103.52%, 98.05%, and 91.02%, respectively, whereas those of the PA group were 105.70%, 100.30%, and 95.50%. Under low-concentration NaCl, the retention values of both groups exceeded or approached 100%, indicating that short-term exposure to low-concentration chloride did not cause obvious loss of compressive performance. As NaCl concentration increased, the compressive strength retention gradually decreased, but the decline was smaller in the PA group. Under 15% NaCl, the PA group exceeded the NC group by 4.48 percentage points, indicating that PA modification improved compressive-strength stability at higher chloride concentrations.

When the immersion duration increased to 90 d, compressive strength retention decreased further, indicating that the cumulative effect of NaCl attack increased with time. The compressive strength retentions of the NC group under 5%, 10%, and 15% NaCl were 96.80%, 90.38%, and 80.14%, respectively, whereas those of the PA group were 97.10%, 93.00%, and 89.20%. Under 15% NaCl, the PA group was 9.06 percentage points higher than the NC group, and this difference was clearly larger than that at 28 d. This shows that the protective effect of PA on compressive performance was more pronounced under the coupled action of high concentration and long exposure, effectively slowing the loss of load-bearing capacity caused by chloride attack.

Figure 3b shows the variation in flexural strength retention with NaCl concentration and immersion duration. At 28 d, the flexural strength retentions of the NC group under 5%, 10%, and 15% NaCl were 101.94%, 94.52%, and 84.52%, respectively, whereas those of the PA group were 108.90%, 103.12%, and 97.14%. Compared with compressive strength retention, flexural strength retention decreased more markedly with increasing NaCl concentration, indicating that chloride attack had a more sensitive effect on crack resistance. Under 15% NaCl, the PA group exceeded the NC group by 12.62 percentage points, showing that PA modification strongly inhibited flexural-performance degradation. At 90 d, the flexural strength retention of both groups decreased further. The values of the NC group under 5%, 10%, and 15% NaCl were 93.90%, 85.10%, and 69.90%, respectively, whereas those of the PA group were 94.60%, 88.60%, and 80.30%. Under 15% NaCl, the PA group was 10.40 percentage points higher than the NC group. These results further indicate that long-term exposure to high-concentration NaCl substantially weakened flexural performance, while PA modification helped maintain material integrity and crack resistance.

Taken together, Figure 2 and Figure 3 show that mechanical degradation under NaCl attack exhibited clear concentration and time effects. Low-concentration NaCl may promote strength development in the short term, but with increasing NaCl concentration and immersion time, compressive strength, flexural strength, and their retention ratios all decreased. Compared with the NC group, the PA-modified group showed better stability in both absolute strength and strength retention, especially under 15% NaCl for 90 d. PA incorporation may reduce the migration rate of chloride solution within the material by optimizing the pore structure, increasing matrix compactness, and enhancing matrix continuity, thereby slowing mechanical degradation. These results indicate that PA modification effectively improves the mechanical durability of cement-based grouting materials in chloride environments, particularly under high chloride concentration and long-term service conditions.

### 3.2. Contact Angle

**Figure 4:**
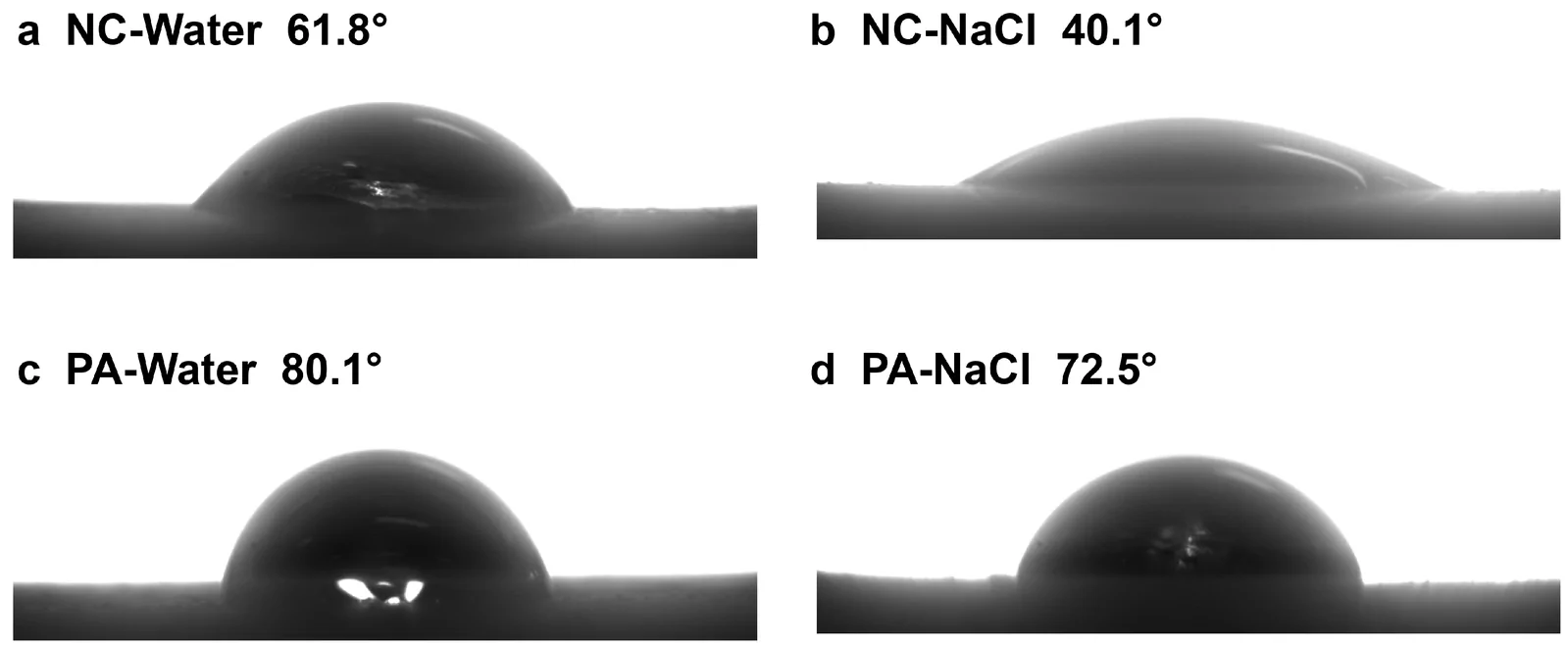
Static water contact-angle images after 90 d of immersion: (**a**) NC-Water; (**b**) NC-15% NaCl; (**c**) PA-Water; and (**d**) PA-15% NaCl.

Contact-angle testing was used to evaluate changes in specimen surface wettability after different exposure conditions. As shown in Figure 4, the contact angle of the NC specimen under water exposure was approximately 61.8°, whereas that of the PA-modified specimen increased to approximately 80.1°, indicating that PA incorporation reduced the wettability of the matrix surface. This result suggests that PA modification may not only improve the internal structure by filling pores, but may also form a relatively low-surface-energy organic phase or continuous film on the material surface, thereby weakening the affinity between the external liquid phase and the matrix surface.

After NaCl exposure, the contact angle of the NC specimen decreased markedly to approximately 40.1°, indicating that salt attack made the unmodified matrix surface more susceptible to wetting. This change may be related to surface roughening, microcrack development, pore exposure, and dissolution of hydration products caused by NaCl attack. Increased surface defects and open pores enhance capillary absorption, allowing the droplet to spread more readily on the material surface. Therefore, the lower contact angle of the NC-NaCl group can serve as surface evidence for reduced attack resistance and is consistent with the decrease in mechanical strength retention described above.

By contrast, the PA-NaCl group retained a contact angle of approximately 72.5° after NaCl exposure. Although this value was lower than that of the PA-Water group, it remained much higher than that of the NC-NaCl group. This indicates that PA modification helped maintain the lower-wettability character of the material surface after salt exposure. The higher contact angle reflects reduced liquid–solid affinity and may decrease the tendency of the external aqueous phase to spread over and enter accessible surface pores. Together with the lower mercury-accessible porosity, improved matrix continuity, and higher mechanical stability of the PA-modified specimens, the contact-angle results support a contribution of reduced surface wetting tendency to improved NaCl resistance.

### 3.3. Pore-Structure Evolution

**Figure 5:**
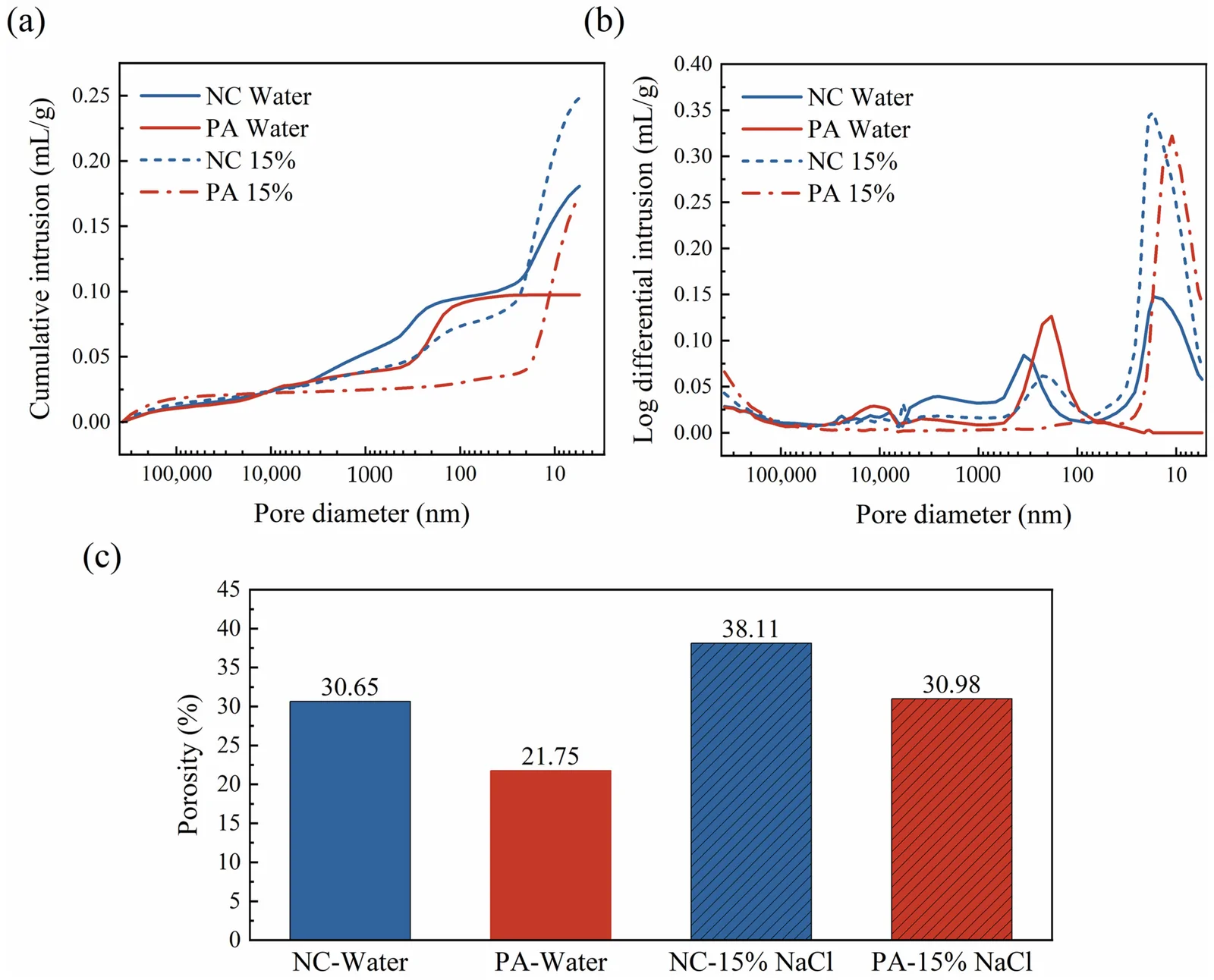
Effect of PA modification on the pore structure after 90 d of NaCl attack: (**a**) cumulative mercury intrusion; (**b**) pore-size distribution; (**c**) porosity.

Figure 5 compares the pore-structure changes in the NC and PA groups under water and 15% NaCl exposure. Under water exposure, both the total mercury intrusion and porosity of the PA-modified group were lower than those of the NC group. The porosity was 30.65% for the NC group and 21.75% for the PA group. This indicates that PA incorporation reduced the pore volume of the cement-based grouting material and made the matrix denser. The pore-size distribution curves also show that the cumulative mercury intrusion of the PA group was generally lower than that of the NC group, suggesting that PA inhibited pore connectivity.

Under 15% NaCl exposure, the porosity of both materials was higher than that of their corresponding water-exposed groups, indicating that high-concentration chloride promoted pore-structure deterioration. The porosity of the NC group increased from 30.65% after water immersion to 38.11%, whereas that of the PA group increased from 21.75% to 30.98%. Compared with the NC group, the PA group still maintained a lower porosity under 15% NaCl, indicating that PA modification slowed the pore connectivity and structural loosening caused by chloride attack.

**Figure 6:**
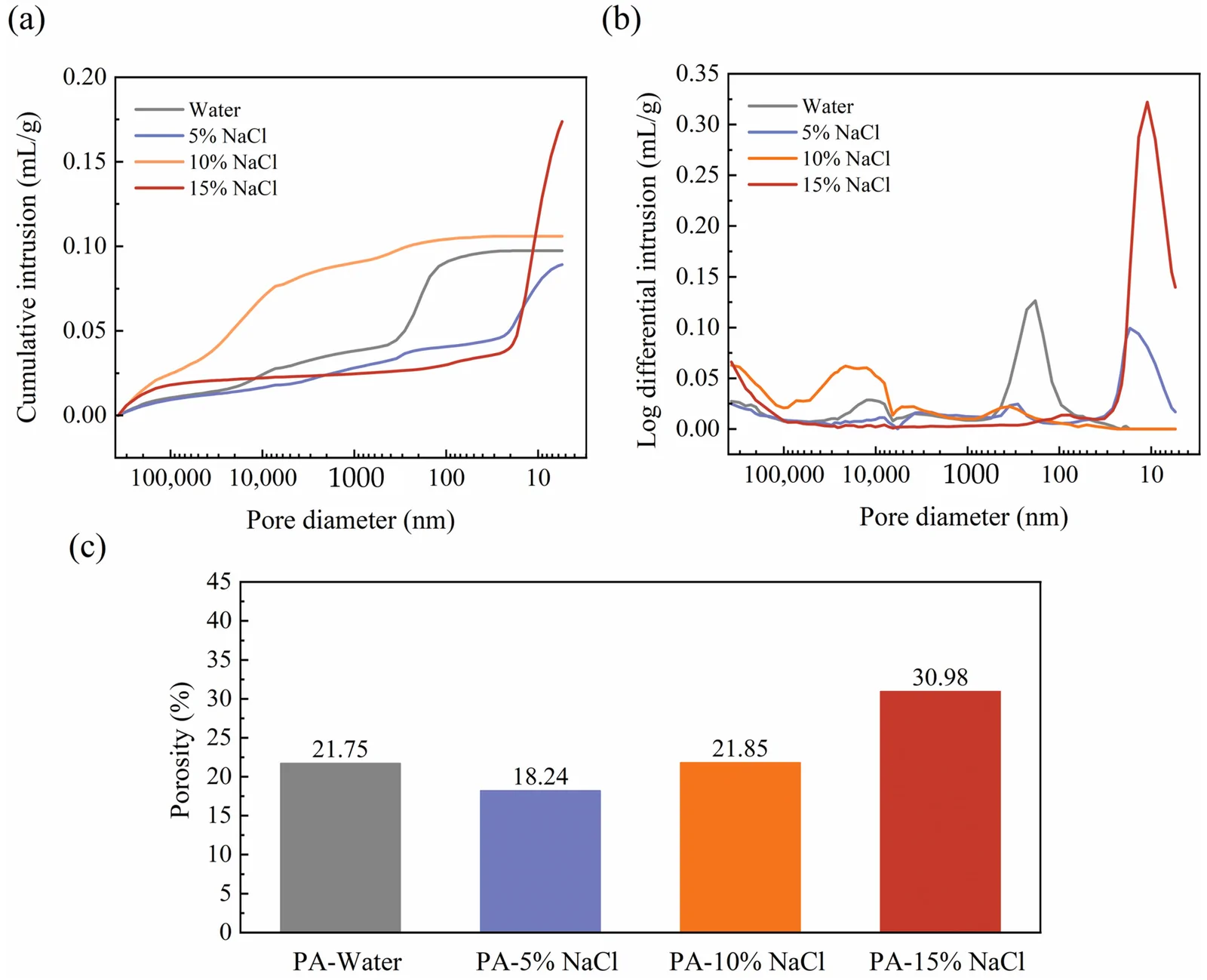
Pore-structure evolution of PA specimens after 90 d of immersion at different NaCl concentrations: (**a**) cumulative mercury intrusion; (**b**) pore-size distribution; (**c**) porosity.

Figure 6 further shows the pore-structure evolution of the PA group under different NaCl concentrations. The porosity of the PA group did not increase monotonically with NaCl concentration, but first decreased and then increased. The porosity was 21.75% under water exposure, decreased to 18.24% under 5% NaCl, increased to 21.85% under 10% NaCl, and further increased to 30.98% under 15% NaCl. This result indicates that low-concentration NaCl did not increase the mercury-accessible pore volume. Instead, the reduced porosity is consistent with a densification response involving continued hydration and/or deposition of chloride-related reaction products within accessible pores.

However, as the NaCl concentration continued to increase, pore-structure deterioration gradually became dominant. Under 10% NaCl, the porosity of the PA group was close to and slightly higher than that of the water-exposed group, but the cumulative mercury intrusion curve rose markedly in the larger-pore region, indicating possible local pore coarsening or enhanced large-pore connectivity. Under 15% NaCl, both total mercury intrusion and porosity reached the highest values among the PA groups, and an obvious intrusion peak appeared in the small-pore region. This suggests that, under high-concentration chloride attack, the filling effect was insufficient to offset attack-induced damage, and pore connectivity and microcrack development may have increased together.

Therefore, the effect of NaCl on the pore structure of PA-modified cement-based grouting materials should be understood as the combined result of product filling and attack-induced damage. Low-concentration NaCl may improve structural compactness by promoting hydration-product formation or pore filling, whereas long-term exposure to high-concentration NaCl may cause pore-wall damage, microcrack propagation, and enhanced pore connectivity, ultimately increasing porosity and total mercury intrusion. PA modification reduced the initial porosity and slowed pore-structure deterioration under high-concentration chloride, although its protective effect could not completely eliminate pore coarsening and the increase in connected pores caused by high-concentration NaCl.

### 3.4. XRD

**Figure 7:**
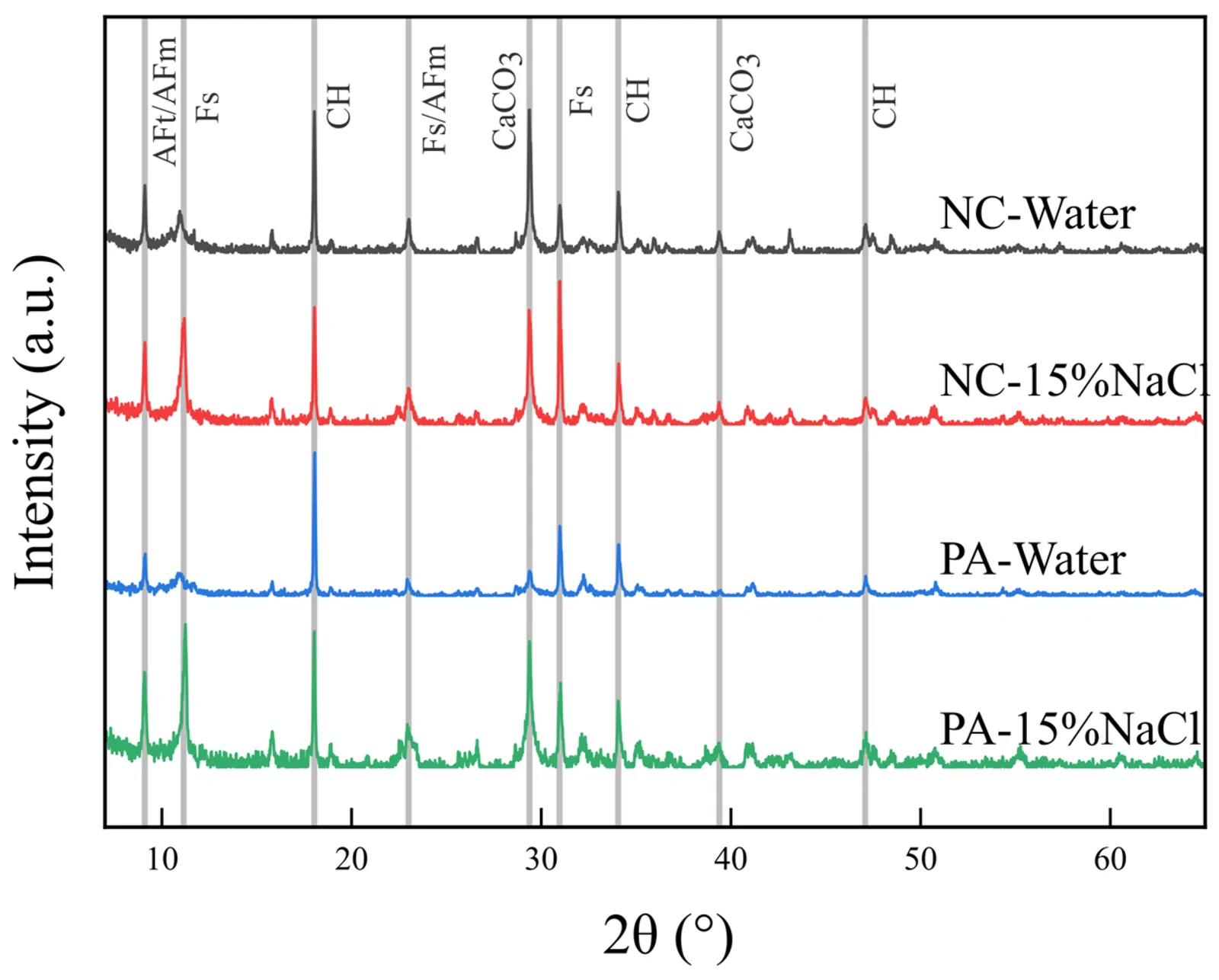
XRD patterns of NC and PA specimens after 90 d of water or 15% NaCl immersion.

Figure 7 illustrates the crystalline-phase evolution of NC and PA-modified specimens after exposure to water and 15% NaCl solution. The water-cured specimens mainly contained portlandite (CH, approximately 18.0°, 34.1°, and 47.1°), calcite (CC, approximately 29.4° and 39.4°), and AFt/AFm-related phases in the low-angle region. After NaCl exposure, clear changes occurred in the AFm-related diffraction region. In particular, the development or enhancement of the reflection near 11.3°, together with supporting diffraction features near 31.1°, is consistent with the formation of Friedel’s-salt-related chloride-bearing AFm phases. Because chloride-bearing AFm phases can coexist with other AFm solid solutions and some reflections may overlap, these peaks are interpreted as evidence of chloride-bearing AFm formation.

Comparison between NC-Water and NC-15%NaCl shows that NaCl exposure promoted the formation of chloride-binding phases in the unmodified matrix. The chloride-associated diffraction features became more pronounced after NaCl exposure, indicating that Al-bearing hydration products participated in chemical chloride binding and AFm phase transformation. Meanwhile, the CH and CC peaks remained clear, showing that NaCl exposure did not simply dissolve the original hydration products, but instead induced a coupled process involving chloride binding, phase transformation, and carbonate-related precipitation. Weak NaCl-related peaks near approximately 31.7° and 45.5° suggest that a small amount of crystalline salt may have remained after drying, but their intensities were lower than those of the main hydration-product peaks.

PA-Water and PA-15%NaCl both retained CH, CC, and AFt/AFm-related hydration products, indicating preservation of the basic cement hydration framework after PA incorporation. The PA-15%NaCl specimen also exhibited chloride-bearing AFm diffraction features after NaCl exposure, demonstrating that chemical chloride binding remained active in the PA-modified matrix. Compared with NC-15%NaCl, the chloride-bearing AFm-related feature near 31.1° was weaker, while the low-angle signal near 11.3° remained detectable. These intensity differences indicate a change in the formation state of the chloride-bearing AFm phases. Because diffraction intensity is influenced by phase abundance, crystallinity, and preferred orientation, the present XRD data do not assign this difference exclusively to a change in Friedel’s salt content.

The improved NaCl resistance of PA-modified specimens should not be interpreted as the disappearance of chloride reactions. Instead, PA modified the coupling between chloride transport and hydration-product transformation. The polymer phase may reduce NaCl-solution ingress and chloride migration into the matrix by increasing surface hydrophobicity and regulating the pore structure, thereby decreasing the amount of effective chloride entering the matrix and participating in reactions, which weakened the crystalline Friedel’s-salt-related phase peak at 31.1°. Meanwhile, residual AFm hydration products could still bind some chloride ions to form chloride-bearing AFm phases, indicating that the PA-modified system retained a certain chloride chemical immobilization capacity. In addition, the formation of chloride-bearing AFm phases and carbonate products may fill pores and refine the microstructure to some extent. Therefore, the XRD results support a multi-mechanism synergistic model in which PA modification improves NaCl resistance through transport inhibition, regulation of chloride binding, and microstructural densification.

### 3.5. TG-DTG Analysis

**Figure 8:**
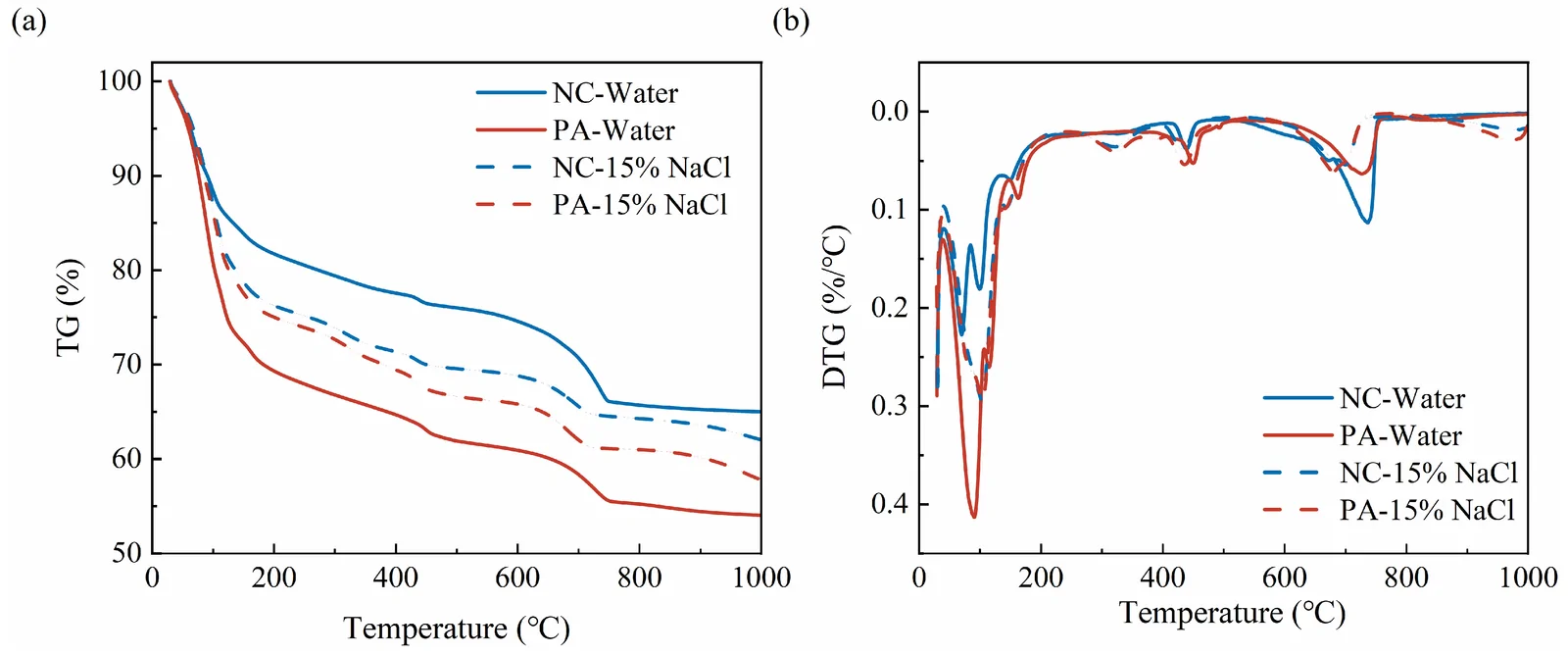
TG-DTG curves of NC and PA specimens after 90 d of water or 15% NaCl immersion: (**a**) TG; (**b**) DTG.

Figure 8 compares the TG-DTG curves of the NC and PA groups under water and 15% NaCl exposure. Overall, the PA-modified group exhibited greater mass loss over the full temperature range. Under water exposure, the total mass loss of the PA group was 45.95%, higher than that of the NC group (34.98%). Under 15% NaCl exposure, the total mass loss of the PA group was 42.17%, also higher than that of the NC group (37.97%). For the PA-containing specimens, the thermal response includes contributions from both the inorganic cement matrix and the PA organic phase. TG studies of waterborne polyacrylate latexes have shown substantial thermal decomposition of the organic polymer component within the temperature range relevant to the present analysis [28]. The TG-DTG results of the PA-containing specimens were therefore interpreted by considering the thermal contributions of retained and bound water, hydration products, carbonate- and chloride-bearing phases, and the PA phase together.

In the low-temperature range of 30–200 °C, the mass loss of the PA-Water group was 30.37%, much higher than that of the NC-Water group (18.02%). This temperature range mainly corresponds to the release of free water, adsorbed water, partial interlayer water, and weakly bound water in hydration products such as C-S-H and AFt. The higher low-temperature mass loss of the PA-modified group may be related to the water-retention effect of the PA polymer, changes in gel-phase content, and retention of more weakly bound water. Meanwhile, the PA group showed lower porosity in the MIP results, indicating that the higher low-temperature mass loss in TG mainly reflects changes in internal water state and the form of bound water in hydration products rather than increased porosity.

After 15% NaCl exposure, the total mass loss of the NC group increased from 34.98% to 37.97%, whereas that of the PA group decreased from 45.95% to 42.17%. This change indicates that NaCl attack affected the two materials differently. For the NC group, NaCl exposure may have promoted partial hydration or formation of salt-related products, increasing the low-temperature mass loss from 18.02% to 23.74%. At the same time, the high-temperature mass loss at 600–800 °C decreased from 8.89% to 4.53%, reflecting changes in carbonates or related high-temperature decomposition components. For the PA group, the low-temperature mass loss decreased from 30.37% to 24.71% after 15% NaCl exposure, but the mass loss in the 400–500 °C range remained relatively high. This suggests that chloride exposure may have reduced part of the weakly bound water or polymer-related mass loss, while still retaining certain Ca(OH)_2_ or related hydration-product decomposition features. It should be noted that the thermal mass-loss range of Friedel’s salt and other chloride-binding products overlaps with those of C-S-H, AFm/AFt, and adsorbed water. Therefore, TG-DTG can only serve as supporting evidence, and the formation of these products should still be confirmed by phase analysis such as XRD.

**Figure 9:**
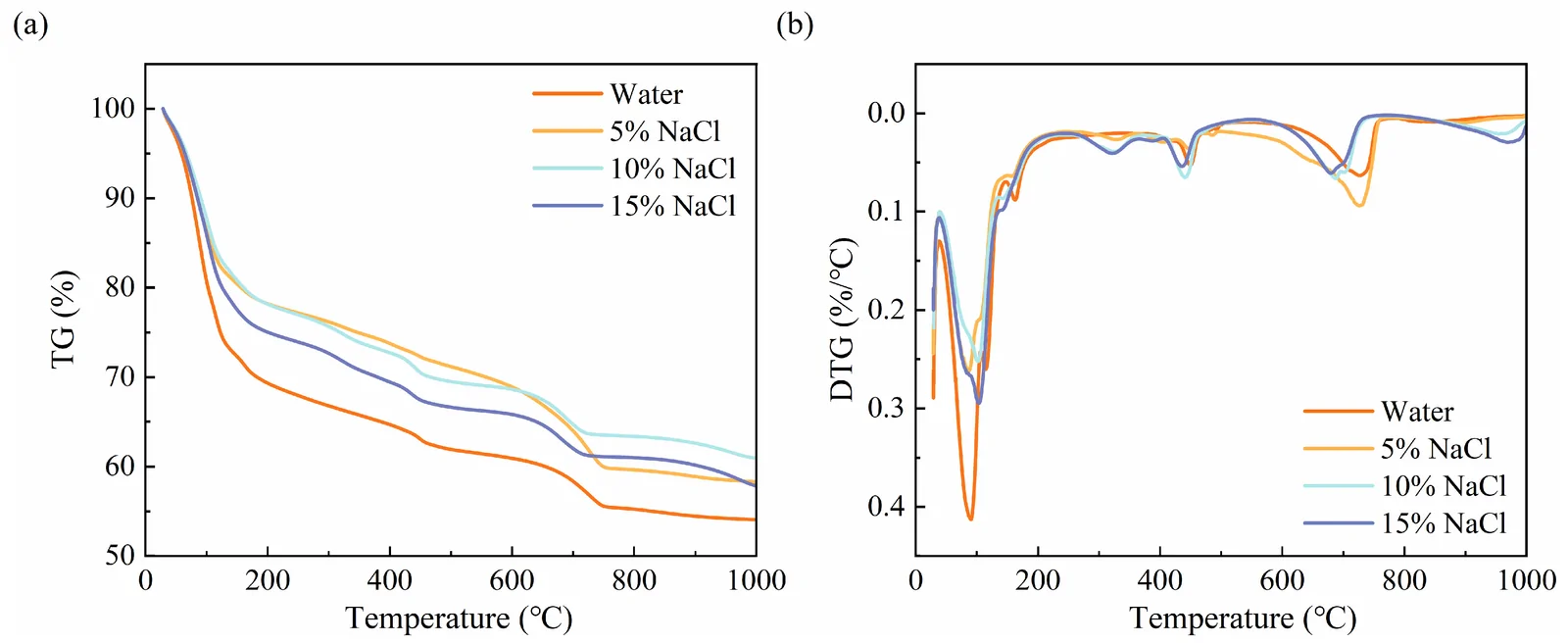
TG-DTG curves of PA specimens after 90 d of immersion at different NaCl concentrations: (**a**) TG; (**b**) DTG.

Figure 9 shows the TG-DTG evolution of the PA-modified group under different NaCl concentrations. The total mass loss of the PA group first decreased and then increased with NaCl concentration, decreasing from 45.95% under water exposure to 41.72% under 5% NaCl and 39.06% under 10% NaCl, then increasing to 42.17% under 15% NaCl. This trend indicates that NaCl has a dual effect on PA-modified materials. At low to moderate NaCl concentrations, the TG-DTG response is consistent with changes in the internal water state and reaction-product assemblage accompanying matrix densification. Together with the MIP results, these changes support contributions from continued hydration and/or reaction-product filling. Under high-concentration NaCl, attack-induced damage, enhanced pore connectivity, and rearrangement of reaction products gradually became dominant, causing total mass loss to increase again.

The results for different temperature ranges further support this interpretation. The low-temperature mass loss of the PA group at 30–200 °C decreased from 30.37% under water exposure to 21.46% and 21.75% under 5% and 10% NaCl, respectively, indicating that low-concentration chloride may reduce weakly bound water release or alter the water state of the gel phase. Under 15% NaCl, this value increased to 24.71%, indicating that the internal water state or microstructural connectivity increased again under high-concentration attack. The mass loss in the 400–500 °C range reached 3.22% under 10% NaCl, higher than that of the water group (2.78%), and may be related to changes in Ca(OH)_2_ content or related hydration products. In the 600–800 °C range, the 5% NaCl group showed a mass loss of 9.29%, markedly higher than the other PA groups, suggesting that a larger fraction of carbonate-containing phases may have formed or been retained under 5% NaCl.

Therefore, the TG-DTG results should be interpreted together with the MIP results. MIP mainly reflects mercury-accessible pores, and pore-size distribution is susceptible to ink-bottle effects and pore-channel connectivity; thus, it is more suitable for comparing relative pore-structure changes among specimens [29,30,31]. Under low-concentration NaCl, the reduced mercury-accessible porosity of the PA-modified material is consistent with the changes observed in the TG-DTG curves, indicating coordinated evolution of the internal water state, reaction products and pore structure. Under high-concentration NaCl, the increases in total mass loss and low-temperature mass loss are consistent with enhanced pore connectivity, microcrack development, or rearrangement of attack products. In other words, the microstructural evolution of PA-modified materials under NaCl attack reflects the combined effects of hydration/salt-product filling, polymer water retention and high-concentration attack-induced damage.

### 3.6. FTIR

**Figure 10:**
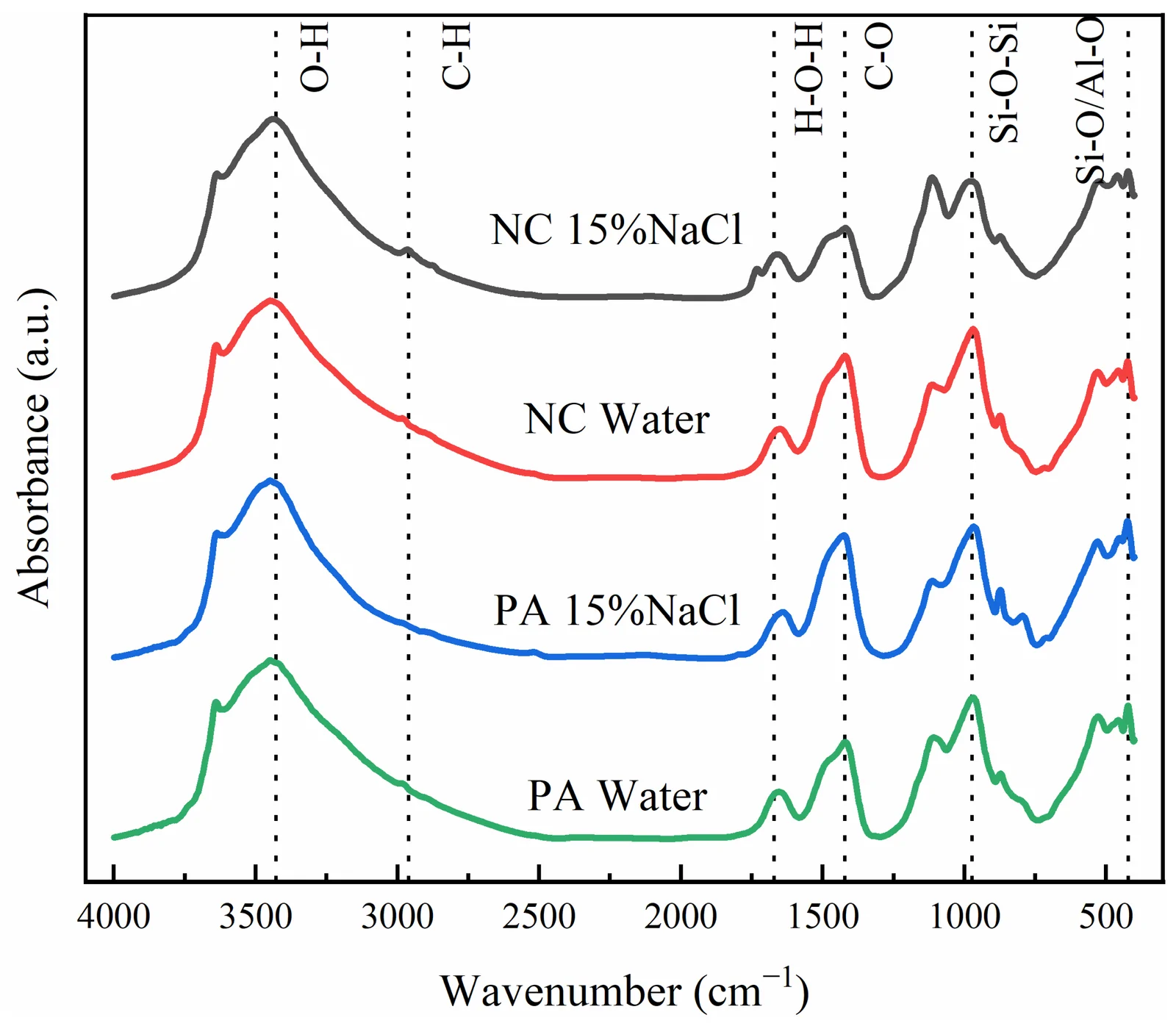
FTIR spectra of NC and PA specimens after 90 d of water or 15% NaCl immersion.

The FTIR spectra in Figure 10 were used to analyze the effects of NaCl attack and PA modification on functional groups and hydration-product structure. All specimens showed a broad O-H stretching band at approximately 3440 cm^−1^ and an H-O-H bending band near 1640 cm^−1^, indicating the presence of bound or adsorbed water after exposure. The absorption bands near 1420 cm^−1^, 870 cm^−1^ and 713 cm^−1^ are mainly attributed to CO_3_^2−^ vibrations, indicating the presence of calcite or other carbonate products. The band near 1120 cm^−1^ is associated mainly with sulfate-related vibrations in sulfate-bearing hydration phases, whereas the band near 970 cm^−1^ is assigned to Si-O stretching in C-S-H or other silicate hydration products. The band near 520 cm^−1^ can be assigned to Si-O/Al-O framework bending vibrations [32,33].

After NaCl attack, NC-15%NaCl showed clear changes in the Si-O-Si and Si-O regions, indicating that chloride exposure affected the local structure of silicate hydration products in the unmodified matrix. Meanwhile, the presence of CO_3_^2−^-related bands indicates the formation or redistribution of carbonate products during NaCl exposure. This is consistent with the appearance of Friedel’s salt/chloride-bearing AFm phases in XRD and the carbonate-related mass-loss changes in TG-DTG, indicating that NaCl attack was not simply salt deposition, but a coupled process involving chloride binding, hydration-product transformation and carbonate-product reconstruction.

The PA-modified specimens retained distinct O-H, CO_3_^2−^, Si-O-Si, Si-O, and Si-O/Al-O characteristic bands after NaCl exposure, indicating that PA incorporation did not destroy the basic hydration-product framework of the cement matrix. The silicate-framework-related absorption bands in PA-15%NaCl remained relatively clear, suggesting that the PA-modified system could maintain a certain degree of hydration-product structural stability in the chloride environment. At the same time, the presence of CO_3_^2−^-related bands in the PA group indicates that carbonate products still participated in phase evolution after exposure and may have jointly affected pore filling and microstructural regulation with chloride-bearing AFm phases.

Taken together with the preceding results, FTIR suggests that PA weakened, to some extent, the attack reaction between cement hydration products and chloride ions. The contact-angle results show that PA reduced specimen surface wettability, which may help delay the ingress of water and NaCl solution. The MIP results show that PA regulated the pore structure and ion-transport pathways. The XRD results show that PA changed the formation state of chloride-binding products, and the TG-DTG results further reflect the reconstruction of hydration products and carbonate products after exposure. Therefore, as supporting evidence, the FTIR results indicate that PA modification may improve NaCl resistance by reducing medium migration, maintaining the silicate hydration-product framework, and regulating the distribution of attack products.

### 3.7. SEM

**Figure 11:**
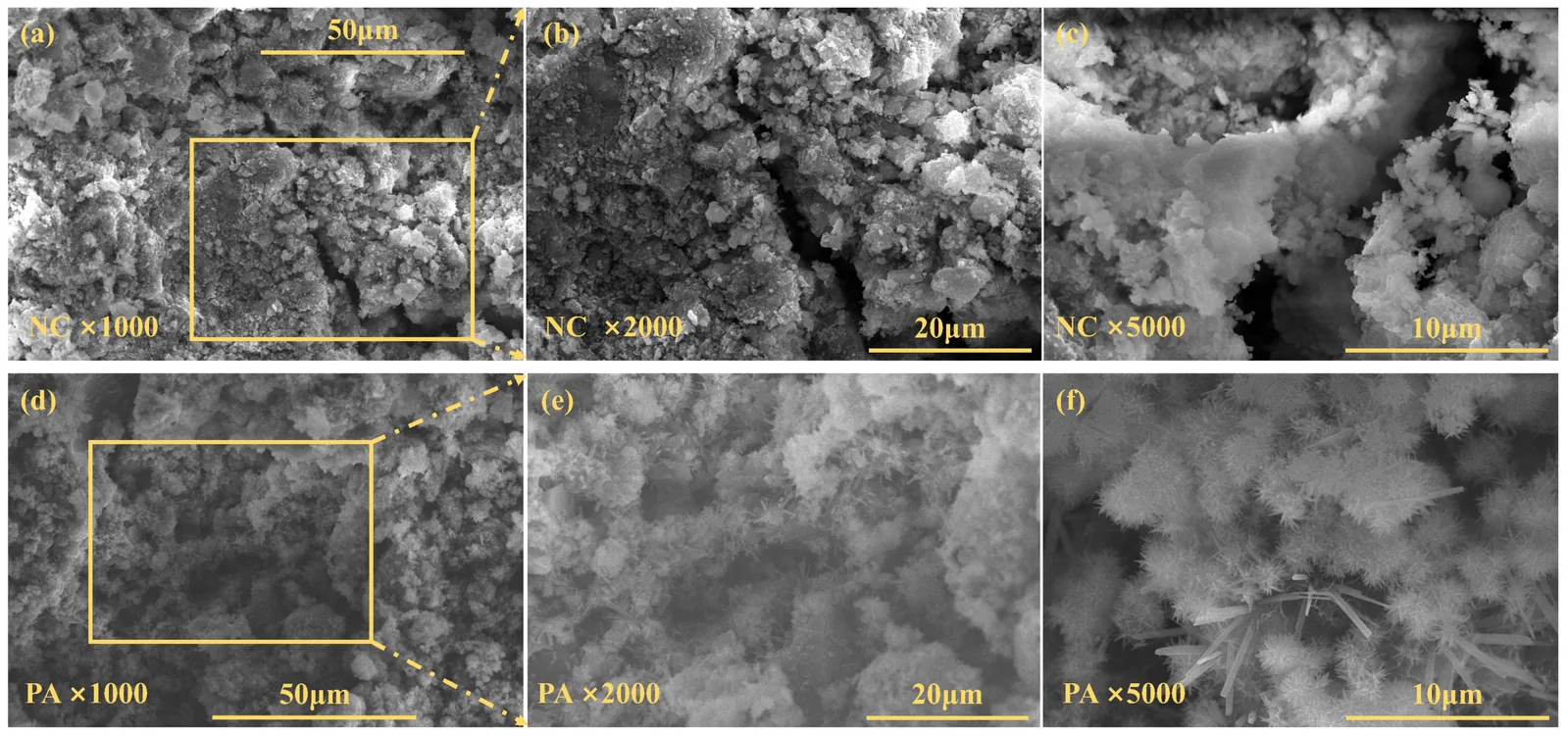
SEM morphology comparison of NC and PA specimens after 90 d of immersion in 15% NaCl: (**a**–**c**) NC group; (**d**–**f**) PA group. Yellow boxes and dotted lines indicate the regions selected for higher-magnification observation.

As shown in Figure 11, SEM observations further reveal the different microstructural responses of NC and PA-modified specimens after NaCl attack. At low magnification, the NC specimen exhibited a loose and discontinuous matrix, with irregular pores and weakly bonded granular products on the surface. Magnified observations show that these pores were locally connected and surrounded by loosely stacked chloride-related reaction products, indicating that NaCl attack had damaged the structural integrity of the unmodified matrix. At higher magnification, product agglomeration and obvious voids were observed in the NC specimen, suggesting that the chloride-related reaction products did not effectively fill the pore network and thus could not form a dense protective structure.

By contrast, the PA-modified specimen exhibited a denser product-filling morphology after NaCl attack. Although local pores and chloride-related reaction products were still observed, the magnified regions showed that these products were mostly embedded in a relatively continuous matrix rather than forming a highly open and separated pore system. Higher-magnification images further show that abundant needle-like and fibrous products crossed and connected local pore regions, indicating that PA modification did not completely suppress chloride-related reactions but changed the spatial distribution of reaction products and their effect on the microstructure. This feature is consistent with the evolution of chloride-related products and silicate/carbonate products observed by XRD and FTIR, and also agrees with the pore-structure regulation reflected by MIP. Together with the higher contact angle of the PA-modified specimens, the SEM analysis supports a coupled protection mechanism: PA reduced NaCl-solution ingress, restricted the development of connected pores, and promoted chloride-related reaction products to form more favorable filling and bridging morphologies, thereby improving the resistance of the matrix to NaCl attack.

### 3.8. Comprehensive Mechanism of NaCl-Attack Resistance in PA-Modified Cement-Based Materials

Combined mechanical, contact-angle, pore-structure, XRD, TG-DTG, FTIR and SEM results show that PA synergistically improved the resistance of cement-based materials in NaCl solution by modifying surface wettability, optimizing pore structure, regulating the formation and distribution of chloride-bearing reaction products, and enhancing matrix bonding and crack resistance.

First, PA incorporation changed the surface wetting behavior of the material. Compared with the NC group, PA-modified specimens had larger contact angles after both water immersion and NaCl attack, indicating that PA helped form a relatively low-surface-energy organic phase on the material surface and some pore walls, thereby reducing the wetting, spreading, and capillary absorption tendency of NaCl solution on the matrix surface. The contact angle of PA-modified specimens after NaCl attack remained 72.5°, markedly higher than that of the NC group (40.1°), indicating that their surface anti-wetting ability was well retained after salt attack. The weaker liquid–solid interfacial affinity could delay the entry of external solution into open pores, providing favorable conditions for reducing subsequent water and chloride migration rates.

Second, PA improved the initial pore structure and reduced the connectivity of the pore network. Under water exposure, PA modification reduced the specimen porosity from 30.65% to 21.75% and also reduced total mercury intrusion, indicating that PA could fill some interparticle voids, improve bonding among hydration products, and decrease the connected pore volume available for liquid transport. After 15% NaCl attack, the porosity and total mercury intrusion of the PA group remained lower than those of the NC group, indicating that PA inhibited pore coarsening, pore interconnection, and microcrack development during attack. After PA regulation, the pore-transport path became more tortuous and less continuous, lengthening the path required for NaCl solution and Cl^−^ to migrate into the material and thereby slowing the accumulation of attack media in the matrix.

After NaCl enters the matrix, Cl^−^ can react with Al-bearing AFm hydration products to form Friedel’s salt or other chloride-bearing AFm phases. XRD results show that chloride-bearing phases were still detected in PA-modified specimens after NaCl exposure, indicating that the modified system retained chemical chloride-binding capacity. Compared with the NC group, the PA-modified specimen exhibited weaker chloride-bearing AFm-related diffraction intensity in the higher-angle region while retaining the characteristic low-angle response. This difference indicates that PA altered the formation state of chloride-bearing AFm phases, while the XRD data alone do not establish the extent of chloride migration. The reduced surface wettability and lower mercury-accessible porosity observed by contact-angle testing and MIP, respectively, provide complementary evidence that PA reduced the accessibility of transport pathways for NaCl solution and Cl^−^. PA may also have affected the nucleation sites, crystallinity, preferred orientation, and spatial distribution of chloride-bearing products. The resulting chloride-bearing AFm phases, carbonate products, and other hydration products can fill local pores and immobilize part of the Cl^−^ to some extent, while the regulation of medium transport and product distribution by PA helps avoid excessive local accumulation of reaction products.

Changes in pore structure and mechanical properties under different NaCl concentrations indicate a competition between low-concentration densification and high-concentration deterioration. Under 5% NaCl, the porosity of the PA group decreased from 21.75% under water exposure to 18.24%, while some early strength values and strength-retention ratios remained slightly higher than or close to the water reference. These results indicate that continued hydration and/or deposition of chloride-related reaction products contributed to pore filling and matrix densification under low-concentration NaCl exposure. As the NaCl concentration and immersion duration increased, pore coarsening, increased pore connectivity, and structural deterioration progressively became dominant. At this stage, newly formed products fill pores and improve matrix compactness. As NaCl concentration and immersion time increase, pore-wall damage, local hydration-product reconstruction, pore connectivity, and microcrack propagation gradually intensify. Consequently, porosity and total mercury intrusion increase, and mechanical properties decline.

Under high-concentration and prolonged NaCl exposure, preservation of matrix continuity became increasingly important to mechanical stability. SEM observations showed that the PA-modified specimens retained a relatively dense and continuous matrix, whereas the NC group exhibited more open pores, loose particles, and connected defects. The PA organic phase, together with the inorganic hydration products, contributed to a more continuous composite microstructure and improved interparticle and interfacial bonding. These microstructural characteristics are consistent with the higher flexural strength and flexural-strength retention observed for the PA-modified specimens.

FTIR results show that PA-modified specimens retained relatively clear O-H, Si-O-Si, Si-O, and Si-O/Al-O characteristic absorption bands after NaCl attack, indicating good stability of the main silicate hydration-product framework. The changes in low-temperature bound water, Ca(OH)_2_, carbonates, and chloride-bearing hydration products reflected by TG-DTG further indicate that PA affected the internal water state, hydration-product composition, and reconstruction of attack products.

Overall, PA slowed the microstructural deterioration caused by NaCl attack by improving surface anti-wetting ability, reducing porosity and pore connectivity, lengthening chloride migration paths, regulating the formation and distribution of chloride-bearing products, and enhancing matrix bonding and crack-propagation resistance. Under low-concentration and short-term conditions, hydration promotion and reaction-product filling helped improve material compactness. Under high-concentration and long-term conditions, attack-induced damage gradually intensified, but PA still reduced the rate of pore coarsening and microcrack development by restricting medium migration, maintaining hydration-product structural stability and improving matrix continuity. Therefore, PA modification delayed the expansion and accumulation of NaCl attack within cement-based materials, made the damage distribution more dispersed, and improved mechanical stability and durability under long-term chloride environments.

The applicability of the present findings is primarily associated with the investigated material system and sustained NaCl exposure under controlled immersion conditions. The results obtained for P·O 32.5 cement with a water-to-cement ratio of 0.80 and a PA-emulsion dosage of 12% provide a comparative basis for evaluating the contribution of PA modification to chloride resistance. In engineering applications, variations in cement composition, water-to-cement ratio, polymer dosage, and admixture system may influence hydration, pore structure, and polymer–cement interactions. Environmental factors such as wetting–drying cycles, temperature fluctuations, flowing groundwater, mixed salt exposure, and mechanical loading can further affect moisture transport, chloride accumulation, and deterioration kinetics. Longer service periods may also involve continued hydration, polymer aging, phase evolution, and progressive microstructural damage. In addition, field performance can be influenced by grout placement, curing quality, structural dimensions, and site-specific exposure conditions. The present results therefore provide a controlled laboratory basis for the engineering application of PA-modified grouting materials under chloride exposure and for subsequent durability evaluation under coupled and prolonged service conditions.

## 4. Conclusions

This study systematically evaluated the mechanical and microstructural response of cement-based grouting materials modified with 12% PA emulsion by mass of cement under continuous immersion in 0–15% NaCl solutions for 28 and 90 d. Based on the integrated mechanical, surface-wettability, pore-structure, phase-composition, and microstructural results, the following conclusions were obtained:

1. PA improved the mechanical stability of the cement-based grouting material under NaCl exposure. After 90 d of immersion in 15% NaCl, the compressive and flexural strengths of the PA group were 23.60% and 22.53% higher than those of the NC group, respectively, while the corresponding strength-retention ratios were higher by 9.06 and 10.40 percentage points.
2. PA reduced surface wettability and maintained a less accessible pore structure during NaCl exposure. After 90 d in 15% NaCl, the contact angle of the PA group was 72.5°, compared with 40.1° for the NC group, while the mercury-accessible porosity was 30.98% and 38.11%, respectively. Within the PA series, porosity decreased from 21.75% under water exposure to 18.24% under 5% NaCl and then increased to 30.98% under 15% NaCl, indicating a concentration-dependent transition from low-concentration densification associated with continued hydration and/or reaction-product filling to pore-structure deterioration at higher NaCl concentrations.
3. NaCl exposure induced chloride-bearing AFm phases consistent with Friedel’s-salt-related products in both NC and PA specimens, confirming that chemical chloride binding occurred in both systems. The distinct diffraction response of the PA group indicates altered formation characteristics of these chloride-binding phases. FTIR results showed that the main silicate hydration-product framework remained identifiable after exposure, while SEM observations revealed a denser and more continuous matrix with fewer open defects in the PA-modified specimens. Together, these results show that PA improved NaCl resistance through the combined regulation of surface wettability, pore structure, chloride-related phase evolution, and matrix integrity.

## Figures and Tables

**Table 1:** Main chemical composition of the cement.

Component	CaO	SiO_2_	Al_2_O_3_	MgO	Fe_2_O_3_	SO_3_
Content (wt.%)	63.00	21.23	6.58	1.60	3.65	2.93

**Table 2:** Basic properties of the PA emulsion.

Property	Appearance	pH	Viscosity (mPa·s)	Solid Content (%)
Measured value	Milky white liquid	7.0–9.0	100–500	76

**Table 3:** Mix proportions of the cement-based grouting materials.

Group	Water/Cement	PA Emulsion/Cement	Cement (g)	PA Emulsion (g)	Added Water (g)
NC	0.8	0.0	100	0	80.00
PA	0.8	0.12	100	12	77.12

## Data Availability

The data presented in this study are available from the corresponding author upon reasonable request.
